# Implementation of Precision Oncology for Patients with Metastatic Breast Cancer in an Interdisciplinary MTB Setting

**DOI:** 10.3390/diagnostics11040733

**Published:** 2021-04-20

**Authors:** Elena Sultova, C. Benedikt Westphalen, Andreas Jung, Joerg Kumbrink, Thomas Kirchner, Doris Mayr, Martina Rudelius, Steffen Ormanns, Volker Heinemann, Klaus H. Metzeler, Philipp A. Greif, Anna Hester, Sven Mahner, Nadia Harbeck, Rachel Wuerstlein

**Affiliations:** 1 Department of Obstetrics and Gynecology and CCC Munich LMU University Hospital, Ludwig Maximilians University (LMU), 81377 Munich, Germany; elena.sultova@med.uni-muenchen.de (E.S.); anna.hester@med.uni-muenchen.de (A.H.); Sven.Mahner@med.uni-muenchen.de (S.M.); Nadia.Harbeck@med.uni-muenchen.de (N.H.); 2 Department of Internal Medicine III and CCC Munich LMU University Hospital, Ludwig Maximilians University (LMU), 81377 Munich, Germany; Christoph_Benedikt.Westphalen@med.uni-muenchen.de (C.B.W.); Volker.Heinemann@med.uni-muenchen.de (V.H.); klaus.metzeler@med.uni-muenchen.de (K.H.M.); Philipp.Greif@med.uni-muenchen.de (P.A.G.); 3 Institute of Pathology and CCC Munich LMU University Hospital, Ludwig Maximilians University (LMU), 81377 Munich, Germany; andreas.jung@med.uni-muenchen.de (A.J.); Joerg.Kumbrink@med.uni-muenchen.de (J.K.); Thomas.Kirchner@med.uni-muenchen.de (T.K.); doris.mayr@med.uni-muenchen.de (D.M.); Martina.Rudelius@med.uni-muenchen.de (M.R.); Steffen.Ormanns@med.uni-muenchen.de (S.O.); 4 Gynecologic Oncology Center and CCC Munich LMU University Hospital, Ludwig Maximilians University (LMU), 81377 Munich, Germany; 5 Breast Center and CCC Munich LMU University Hospital, Ludwig Maximilians University (LMU), 81377 Munich, Germany

**Keywords:** precision oncology, personalized medicine, metastatic breast cancer, molecular tumor board, molecular diagnostics

## Abstract

The advent of molecular diagnostics and the rising number of targeted therapies have facilitated development of precision oncology for cancer patients. In order to demonstrate its impact for patients with metastatic breast cancer (mBC), we initiated a Molecular Tumor Board (MTB) to provide treatment recommendations for mBC patients who had disease progression under standard treatment. NGS (next generation sequencing) was carried out using the Oncomine multi-gene panel testing system (Ion Torrent). The MTB reviewed molecular diagnostics’ results, relevant tumor characteristics, patient’s course of disease and made personalized treatment and/or diagnostic recommendations for each patient. From May 2017 to December 2019, 100 mBC patients were discussed by the local MTB. A total 72% of the mBC tumors had at least one molecular alteration (median 2 per case, range: 1 to 6). The most frequent genetic changes were found in the following genes: *PIK3CA* (19%) and *TP53* (17%). The MTB rated 53% of these alterations as actionable and treatment recommendations were made accordingly for 49 (49%) patients. Sixteen patients (16%) underwent the suggested therapy. Nine out of sixteen patients (56%; 9% of all) experienced a clinical benefit with a progression-free survival ratio ≥ 1.3. Personalized targeted therapy recommendations resulting from MTB case discussions could provide substantial benefits for patients with mBC and should be implemented for all suitable patients.

## 1. Introduction

Breast cancer is both the most common malignancy and the leading cause of cancer-related death among women worldwide, with an estimated 2,088,849 new cases and 626,679 deaths in 2018 [1]. Diagnostic and treatment options have progressed substantially over the past few years, which led to slightly increasing incidence rates and a decline in breast cancer mortality [2,3]. However, despite recent advances in oncology over the past few years, not all patients equally benefit from these improvements. Survival rates of patients with metastatic breast cancer (mBC) remain very poor compared to those of breast cancer patients at earlier disease stages. While patients with a localized or regionally confined breast cancer have a 5-year relative survival rate of 99% and 86%, mBC remains an incurable disease with a median overall survival of approximately 3 years and a 5-year survival of only 27% [4,5]. Moreover, as still 20–30% of breast cancer patients diagnosed at an early stage are likely to develop metastatic disease during the course of their disease [6], it is essential to develop new treatment concepts for this group of patients.

**Figure 1:**
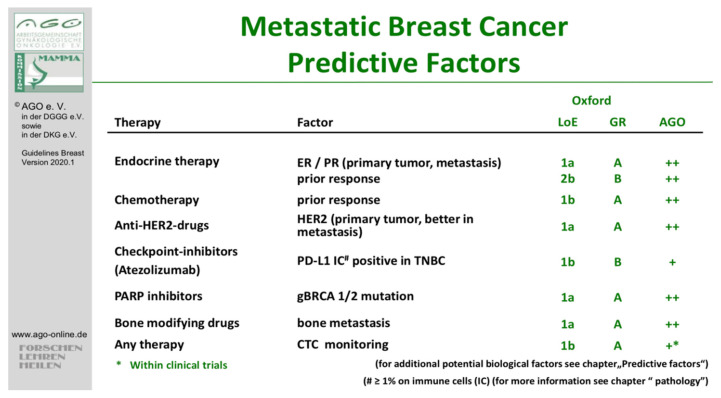
Predictive factors in metastatic breast cancer (mBC), German Gynecological Oncology Group (http://www.ago-online.de assessed on 21 February 2021). (ER = estrogen receptor, PR = progesterone receptor, HER2 = human epidermal growth factor, PD-L1 = programmed death-ligand 1, TNBC = triple-negative breast cancer, PARP = poly (ADP-ribose) polymerase, gBRCA = germline BRCA, CTC = circulating tumor cell, LoE = levels of evidence, GR = grade, AGO = Arbeitsgemeinschaft Gynäkologische Onkologie (German Gynecological Oncology Group).

Recent technological advances in DNA sequencing have promoted discovery of biomarkers or oncogenic drivers that provide new treatment strategies for patients lacking other therapy alternatives. Biomarker analysis is a routine practice in breast cancer. Historically, estrogen receptor (ER) and progesterone receptor (PR) have been successfully used as predictive biomarkers for endocrine therapy [7]. Moreover, such biomarkers not only provide information about patients’ response to a particular treatment but also have a prognostic value. For instance, several studies demonstrated that patients with ER or PR-positive tumors tend to have a better outcome than those lacking these receptors [8,9,10,11]. Recently, a heightened interest in the relevance of biomarkers in oncology has been witnessed, as their potential for guiding treatment decisions has been recognized. In the past years, impressive advances in cancer treatment outcomes through the combined use of molecular diagnostics and targeted therapies have been seen in various tumor entities [12,13,14,15]. For breast cancer patients, a rising number of predictive biomarkers have led to development of several new drugs designed for targeting specific genetic alterations, such as PARP (poly(ADP-ribose)-polymerase) inhibitors like olaparib or talazoparib for germline *BRCA*-mutated breast cancer [16] (Figure 1).

Moreover, since the introduction of anti-*HER2* targeted agents, survival rates of patients with *HER2*-positive mBC have remarkably improved [17,18,19]. Patients with *HER2*-positive disease, who received the anti-*HER2* agent trastuzumab, had a 44% decreased risk of death compared to the control group, which has turned trastuzumab into a routinely used drug today [20]. Besides *HER2* amplification, *HER2* mutations have become a predictive marker as well. Responses to neratinib, a tyrosine kinase inhibitor, were seen in about 30% of patients with *HER2* mutations. Moreover, when combined with fulvestrant in previously treated hormone receptor-positive *HER2*-mutated tumors, it showed responses in the range of 40% [21]. Recently, multiple targeted agents have become available that have improved the outcomes of patients with breast cancer, with alpelisib being the most recent example. It proved to be beneficial for patients with a *PIK3CA*-mutated breast cancer, thus adding the *PIK3CA* gene to the list of ESCAT Level 1 actionable mutations. Patients treated with alpelisib had a progression-free survival (PFS) of 11 months (95% confidence interval [CI], 7.5 to 14.5), as compared with 5.7 months (95% CI, 3.7 to 7.4) in the control arm (hazard ratio for progression or death, 0.65; 95% CI, 0.50 to 0.85; *p* < 0.001) [22].

The advent of multiple targeted therapeutics and the promising advances in DNA sequencing techniques promoted research on molecular tumor characteristics and led to development of a new approach now known as “precision medicine”. Its major aim is to use targetable molecular alterations for identification of specific subpopulation of patients, whose tumors express these markers and therefore could benefit from a certain treatment.

However, there is still a lack of clinical data on the impact of implementing precision medicine for patients with mBC (Table 1). In order to evaluate whether this subset of patients could benefit from this new approach, we initiated a molecular tumor board (MTB) to give personalized treatment recommendations based on comprehensive molecular tumor profiling. Here, we present the results of the first 100 mBC patients discussed at the Comprehensive Cancer Center, LMU Munich Molecular Tumor Board.

## 2. Materials and Methods

### 2.1. Patient Recruitment and Study Design

A total of 100 patients from Breast Center LMU were enrolled in a prospective single-center registry “The Informative Patient”, conducted at the LMU University Hospital Munich in cooperation with the Comprehensive Cancer Center Munich. Informed consent was obtained from all individual participants. The registry was approved by the ethics committee of the LMU University Hospital Munich (reference number: 284-10). The study protocol was in accordance with the Declaration of Helsinki. The population presented here were accrued between May 2017 and December 2019. Key inclusion criteria were as follows: histological confirmation of breast cancer disease, at least one metastatic site, Eastern Cooperative Oncology Group (ECOG) performance status of 0 or 1, and willingness to take part in potential clinical trials or to start an off-label treatment. Molecular diagnostic testing was performed at the Pathology Institute of the LMU University Munich. The primary objective of the study was to use personalized recommendations made by a multidisciplinary tumor board to improve the progression-free survival compared to the previous treatment and to prove the impact of the MTB recommendation on the overall survival of mBC patients. Here, we present an organ-specific analysis of the first 100 patients with metastatic breast cancer, who took part of the “The Informative Patient” study. Details on progression-free survival, as well as on overall survival of all patients, who took part of the study, are yet to be presented.

### 2.2. Panel-Guided Next-Generation Sequencing

Molecular analyses were performed at the LMU Institute of Pathology. Sections from formalin fixed paraffin embedded (FFPE) tissue samples were prepared followed by hematoxylin-eosin staining of the first slide. Appropriate tissue regions were selected, and nucleic acids were extracted from subsequent sections using the GeneRead (DNA) and RNeasy FFPE kits (RNA) (both from Qiagen, Hilden, Germany). Targeted NGS was performed with the Oncomine Focus Panel (covering 52 cancer-associated genes) till November 2018 and then with the Oncomine Comprehensive v.3 assay screening for genetic alterations in 161 cancer-associated genes at the levels of DNA (single-nucleotide variants (SNV), multi-nucleotide variants (MNV), small ins, del, indels, copy number variation (CNV)) and RNA (gene fusions). Briefly, libraries were generated employing Ampliseq Library Plus-, Ampliseq cDNA synthesis-, Ampliseq CD index, Ampliseq Equalizer- together with AmpliSeq for Illumina Comprehensive Panel v3 (all Illumina) or Oncomine Comprehensive Assay v3 and Ion AmpliSeq Library-, IonXpress Barcode Adapter-, Ion Library Equalizer-kits together with Ion Chip kits (540 and 550) (all Thermo Fisher, Waltham, MA, USA) following each step of the respective user manuals. Libraries were sequenced on an Ion Torrent GeneStudio S5 Prime (Thermo Fisher) or Illumina 500 Next Seq (Illumina) next generation sequencing (NGS) machine. Analysis of the results was performed with either the Ion Reporter System (Thermo Fisher) followed by further variant and quality interpretation with a home-made excel tool or the Illumina Local Run Manager with subsequent annotation of VCF-files using wAnnovar [23] and a home-made python-script filtering for clinically relevant mutations. Alterations were confirmed with the Integrated Genomics Viewer (IGV, Broad Institute, Cambridge, MA, USA) Mutations were judged as relevant on the basis of the interpretation criteria utilized in ClinVar [24]. Only likely pathogenic and pathogenic mutations as well as VUS (variant of unknown significance or not evaluated in ClinVar with a prediction trend of being likely pathogenic—majorly frameshift or truncating variants) with allele frequencies ≥3% were reported. A comprehensive pathological report comprising NGS results together with data from immunohistochemistry (used for HER2 and PD-L1 testing), fluorescence in situ hybridization (FISH) (used for confirming of the HER2 status) and histo-morphology was submitted to the MTB for further discussion of therapeutic options.

### 2.3. Study Procedure

**Figure 2:**
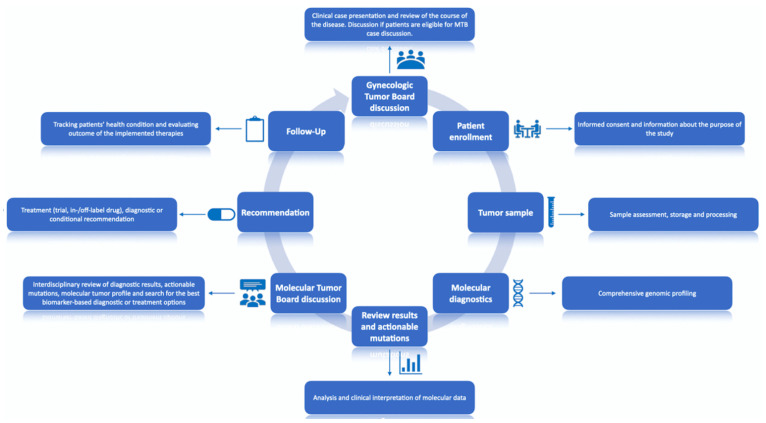
“The Informative Patient” study design. All procedures were conducted in the LMU University Hospital, Munich.

A flowchart of the trial “The informative patient” is shown in Figure 2.

All patients (n = 100) were first discussed in an organ-specific breast cancer tumor board (LMU, Department of Obstetrics and Gynecology), where the treating gyneco-oncologist presented the patient’s case and requested case discussion at the Molecular Tumor Board (MTB). If eligible, after patient informed consent, all tumors underwent comprehensive molecular profiling and the results were then presented to the MTB. Each case was then discussed by the multidisciplinary MTB team, consisting of gyneco-oncologists with expertise in various cancer entities along with molecular pathologists, and genetic counselors. Each patient was presented by a moderator, who provided information about patient’s course of disease, prior treatment history with response and comorbidities. After reviewing clinical history and molecular profile of each tumor, the MTB discussed actionability of the discovered mutations by reviewing literature and publicly available databases, such as PubMED, clinicaltrials.gov accessed on 10 April 2021, ClinVar, Varsome, OncoKB and CIViC [25]. The purpose of this research was to determine frequency of particular molecular alterations across patient populations as well as relevant pathways that may be affected, and then matching them to available drugs (in- and off-label) or clinical trials. For each patient, the MTB discussed possible diagnostic and treatment options and issued recommendations accordingly. Treatment recommendations were supported by levels of evidence for molecular targets by using the European Society for Medical Oncology (ESMO) Scale for Clinical Actionability of Molecular Targets (ESCAT), defined according to their implications for patient management (Table 2) [26].

### 2.4. Analysis of Results

In order to determine the clinical impact of panel-guided NGS adjusted therapies, we calculated progression-free survival ratio (PFSr) as previously described by Von Hoff et al. [28], by comparing progression-free survival on matched therapy (PFS2) with progression-free survival on the most recent therapy prior to NGS testing on which the patient experienced disease progression (PFS1). Progression-free survival (PFS) was calculated from start of recommended treatment to disease progression (as assessed by RECIST guidelines (version 1.1) or death whatever occurred first) [29]. Cut-off date for follow-up analysis was 1 August 2020.

## 3. Results

### 3.1. Patient Characteristics

Between May 2017 and December 2019, 100 female mBC patients were included in the “The Informative Patient” study. The median age was 52 years (range: 30 to 82). Patients had a median of four therapies prior to inclusion (range: 1 to 13). The median number of metastatic sites per patient was 2 (range: 1 to 6). Regarding organ sites, the majority of patients had bone metastases (62%), followed by liver (51%), and lung (40%) metastases (Supplementary Table S2.)

More information about patients’ characteristics is listed in Table 3.

**Figure 3:**
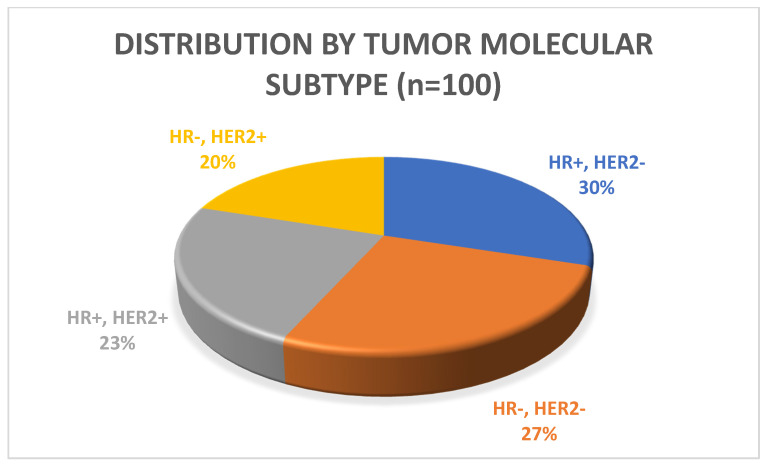
Patient distribution by tumor molecular subtype (by immunohistochemistry (IHC) (n = 100), (HR = hormone receptor, *HER2* = human epidermal growth factor)**.**

The plurarity of patients had triple-negative breast cancer (ER, PR and *HER2* negative; n = 30; 46.9%), followed by estrogen receptor (ER)—positive and/or progesterone receptor (PR)—positive, human epidermal growth factor receptor 2 (*HER2*)—negative (luminal-like) (n = 28; 43.8%), or *HER2*-positive, ER-negative, PR-negative disease (n = 5; 7.8%) at time of the initial MTB presentation; one patient (1.6%) had triple-positive disease (ER-positive, PR-positive and *HER2*-positive) (Figure 3).

### 3.2. Molecular Diagnostics

NGS was done for all patients. All tissue samples were collected either prior to molecular profiling or prior to the initiation of the last therapy a patient received. When selecting appropriate tissues, we set the criteria of using samples that were not older than two years prior to initiation of molecular profiling, and when possible, collecting tissue samples after the last standard line of therapy, in order to provide the most accurate analysis of molecular profile data.

All tumor samples used for molecular profiling have been collected no more than 24 months prior to molecular profiling. The median turnaround time for completing molecular profiling was 19 days (range: 10–48). The median turnaround time between initiation of molecular profiling and MTB case discussion was 33 days, which is similar to reported median turnaround times in other studies [30].

In seven cases (7%) tumor sequencing was performed more than once. In 73 (73%) of the received samples, at least one molecular alteration was found. Among these 73 tumor samples, 53 (53%) had at least one actionable mutation, as classified by the MTB. More than one molecular alteration was found in 51 cases (51%). No genomic alterations were found in 27 samples (27%), 11 of which (11%) had insufficient material quality and therefore led to technically not successful molecular analysis.

**Figure 4:**
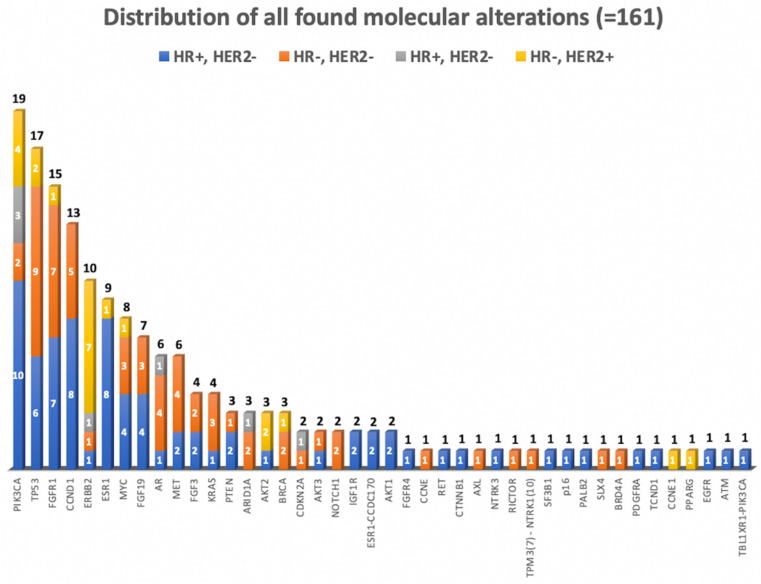
Distribution of genomic alterations sorted by tumor molecular subtype (n = 100) (HR = hormone receptor, *HER2* = human epidermal growth factor).

All in all, we detected 161 molecular alterations, with a median of two alterations per sample (range: 0–6). In total, molecular changes in 42 genes were found. As shown in Figure 4, the most common molecular alterations across the sequenced samples were found in the *PIK3CA* gene (19/100; 19%); followed by *TP53* gene (17/100; 17%), and *FGFR1* gene (15/100; 15%) (Supplementary Table S1).

In our cohort, the most altered oncogenic signaling pathway was the *RTK/RAS* pathway, with 15% *FGFR1* alterations, 10% *ERBB2* and 6% *MET* alterations, followed by the *PI3K/mTOR/AKT* pathway. A total 20% of the patients had a non-actionable mutation, most frequently in the *TP53* gene (17%).

### 3.3. Recommendations

In total, 49 patients (49%) received at least one treatment recommendation from the MTB. Further, 18% of all patients obtained more than one treatment recommendation, as their samples contained more than one actionable alteration. The most common therapy recommendation (in 21 of 49 cases with at least one treatment recommendation) was everolimus, a mTOR inhibitor. Of note, five patients carrying a now actionable mutation (*PIK3CA*, found in 19% of patients in the presented cohort) received no therapy recommendation, as the drug targeting this mutation (alpelisib) was not approved at the time of MTB presentation.

In five of the cases (5%), the MTB suggested further diagnostic tests, three of which then resulted in a treatment recommendation. In the Appendix A, details on actionable mutations and following MTB treatment recommendations made by the MTB are provided.

**Figure 5:**
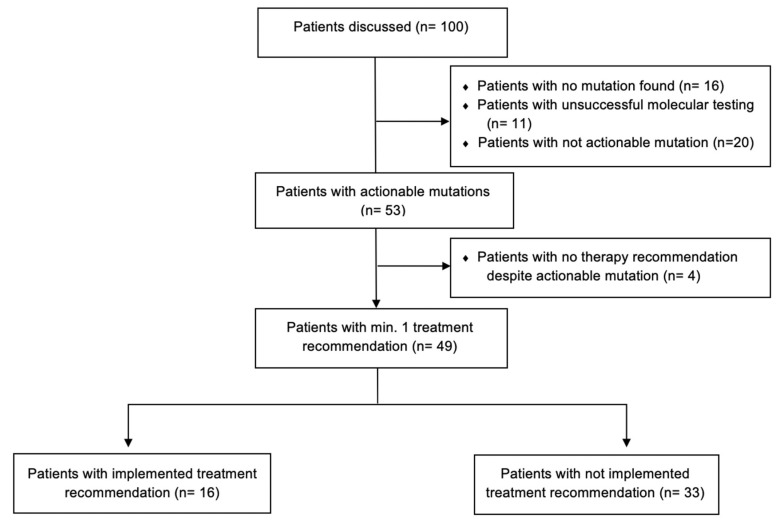
Consort flow diagram showing the results of Molecular Tumor Board (MTB) case discussions based on molecular diagnostics results and implementation of treatment recommendations in our cohort (n = 100).

All in all, 51 patients (51%) received no recommendation from the MTB. The main reasons for no recommendation were absence of molecular alterations in the NGS testing (27%), non-actionable mutations (20%), patient comorbidities or general condition by the time of MTB case discussion (3%). More information about the results of MTB case discussions is listed in Figure 5.

### 3.4. Progression Free Survival Analysis

Follow-up information was available for 48 out of 49 patients with a treatment recommendation. In 16 out of 49 cases (16% of all patients), treatment recommendations were implemented. Lack of implementations was mostly caused by deterioration of the patient’s health condition (10%), inaccessibility to treatment recommendation (8%), not fulfilling trial inclusion criteria (6%), or patient preferences (5%).

The median turnaround time between the discussion in the molecular tumor board and the initiation of recommended therapies was 53 days. Among patients with implemented treatment recommendations, 13 (13%) received an in-label treatment, whereas three (3%) received an off-label drug. The most frequently implemented treatment recommendation was a mTOR inhibitor (mostly everolimus) in combination with endocrine therapy (mostly exemestane) in seven cases (7%). A total 9 of 16 patients (56%, 9% of all patients) with implemented treatment recommendations were found to have a PFSr ≥ 1.3, with a median of 1.3 (range: 0.2 to 11.8). Further, 6 patients (6%) achieved a state of partial remission or stable disease lasting over 16 weeks, with one patient having an ongoing PFS (Table 4).

**Table 4:** Patients with implemented treatment recommendations (in- and off-label).

**#**	**Gene Alteration**	**Implemented Therapy (MTB Recommendation)**	**Previous Therapy**	**Label**	**PFS2 (Weeks)**	**PFS1 (Weeks)**	**PFSr**
1	***FGFR1***	Everolimus [31,32]	Capecitabine	in	14	81	0.2
2	***FGFR1***	Everolimus	Capecitabine/Bevacizumab	in	4	13	0.3
3	***PIK3CA***	Alpelisib (ESCAT I)	Everolimus/Exemestan	in	14	44	0.3
4	***PIK3CA***	Alpelisib (ESCAT I)	Palbociclib/Anastrozol	in	15	32	0.5
5	***FGFR1***	Everolimus	Trastuzumab/Eribulin	In	4	8	0.5
6	***ERBB2***	Trastuzumab/Lapatinib (ESCAT I)	Trastuzumab-Emtansin	in	21	25	0.8
7	***FGFR1***	Everolimus	Eribulin	in	13	13	1
8	***PIK3CA***	Everolimus	Trastuzumab/Pertuzumab	in	69	55	1.3
9	***FGFR1***	Everolimus	Docetaxel/Pertuzumab/Trastuzumab	in	13	9	1.4
10	***PIK3CA***	Everolimus	Paclitaxel	in	18	12	1.5
11	***CCND1***	Palbociclib	Carboplatin/Gemcitabine	in	21	13	1.6
12	***PIK3CA***	Alpelisib (ESCAT I)	Carboplatin/Gemcitabine	in	15	9	1.7
13	***FGFR1***	Pazopanib	Cyclophosphamid/Methotrexat/Fluorouracil	off	12	6	2
14	***FGFR1***	Pazopanib	Eribulin	off	6	3	2 *
15	***ERBB2***	Lapatinib (ESCAT II)	Epirubicin	in	26	3	8.7
16	***p16, MYC***	Pembrolizumab [33,34,35]	Cisplatin/5-Fluorouracil	off	59	5	11.8

PFS1 = progression-free survival on the most previous line of therapy (standard of care). PFS2 = progression-free survival on the implemented recommended therapy. PFSr = PFS ratio = PFS2/PFS1. * Clinically not meaningful result, as PFS1 is too short [36].

**Figure 6:**
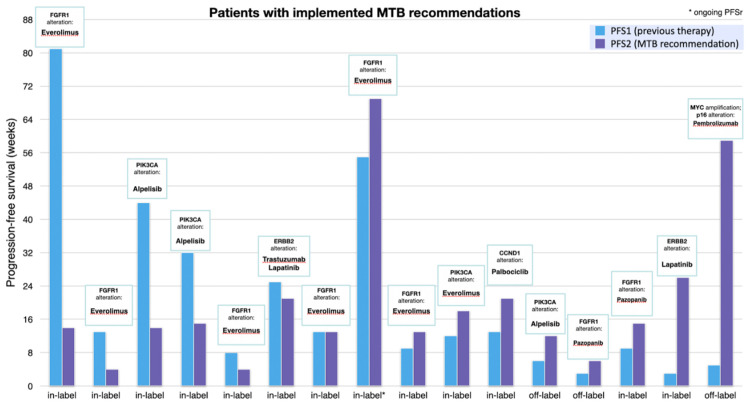
Bar graph comparing progression-free survival (PFS) of previous line of therapy (PFS1) and of implemented therapy, as recommended by the MTB (PFS2). PFS was defined as the period of time between the start of treatment till disease progression or death.

Figure 6 details the actual comparison of PFS on recommended therapy (PFS2) versus PFS on last therapy the patient received (PFS1).

## 4. Discussion

Modern sequencing techniques together with newly targeted therapies have revolutionized cancer medicine by providing substantial benefits for cancer patients in comparison to prior medical standards. However, the precision oncology movement remains controversial, as evidence supporting this approach is still missing. In 2015, a meta-analysis conducted by Schwaederle et al. compared results of 570 studies comprising 32,149 patients, divided in two groups of patients who received a personalized treatment strategy versus those that did not. The results supported the personalized approach, as it correlated with higher median response rate (31% vs. 10.5%), prolonged median PFS (5.9 vs. 2.7 months) and overall survival (13.7 vs. 8.9 months) [37]. Many other studies demonstrated similar positive results [38,39,40,41]. For instance, in the WINTHER trial, 22.4% of the patients receiving therapy based on molecular profiling had a survival ratio > 1.5. However, in the first randomized trial, SHIVA (n = 741 screened) no significant improvement in PFS was seen in the precision oncology arm compared to the standard-of-care arm, suggesting that off-label use of targeted therapies does not improve PFS compared with standard-of-care treatment [42]. All in all, over the past few years, many researchers have investigated the effect of using panel-guided molecular diagnostics on the PFS and OS of patients with advanced cancers. While some of them were able to demonstrate a clinical benefit and longer survival for patients with individualized therapies, the overall impact remains small, and therefore, a subject to discussions of the cost-effectiveness of this approach [43].

In this study, we demonstrated that individual treatment recommendation based on molecular profiling using NGS could improve PFS of mBC patients. Among those patients with implemented treatment recommendations, more than a half had a PFSr ≥ 1.3, which demonstrates the potential relevance of involving targeted NGS-guided therapies in mBC. Previous studies focusing on implementation of precision oncology in breast cancer care also showed similar results, demonstrating that this approach is feasible and of great importance—at least for a subset of patients [44]. For instance, in the SAFIR01 trial, 9% of the patients with implemented treatment recommendation had an objective response, while 21% responded with stable disease lasting more than 16 weeks [45]. Other recent studies, such as the one by Geelen et al., which accrued 357 breast cancer patients of whom 74% had a potentially actionable alteration, also demonstrated feasibility of using molecular diagnostics to detect actionable molecular alterations. This suggests that clinical utility of genomic profiling in combination with more available targeted therapies will expand over time [46].

Within the context of mBC, there are various applications of molecular diagnostics, which could potentially improve patient outcome. Apart from identifying oncogenic driver mutations, it is also possible to define genomic alterations, associated with secondary resistance, another major clinical problem in mBC. For example, *ESR1* mutations, occurring in 10–30% of pre-treated ER-positive mBC, are known to cause resistance to aromatase inhibitors [47]. Thus, detecting such alterations could provide valuable information about signaling pathways causing resistance to certain treatments. As tumor biological factors of breast cancer often tend to differ in the primary and in the distant metastatic tissue, affecting patient prognosis, there is a need of understanding the tumor biology of these malignancies at a higher level [48].

In the presented study, approximately 25% of the patients had a Level 1 actionable alteration, corresponding to ESCAT levels of evidence (LOE) I/II genes, with *PIK3CA* being the most frequently altered gene. The latest breakthrough in breast cancer oncology was the approval of alpelisib in combination with fulvestrant. Of note, some patients in our cohort, harboring a *PIK3CA* mutation, did not receive a treatment recommendation, if the *PIK3CA* gene was not classified as “actionable” at time of their initial MTB presentation. Thus, considering the high frequency of *PIK3CA* gene alterations in breast cancer (more than 25% of all breast malignancies), the discovery of alpelisib was of great importance for many patients, proving that detecting genomic alterations is crucial, as research in the past decade has been mainly focused on developing new drugs targeting such molecular aberrations [49].

However, although the number of MTAs (molecular targeted agents) is constantly rising, there is still a lack of drugs targeting many genes, commonly expressed in breast cancer, like *TP53* mutations (17% of our patients expressed this alteration) for example, making matching genomic alterations with targeted therapies still a great challenge for the majority of patients and one of the greatest limitations for precision oncology. Developing newly targeted therapies represents a major issue, as it requires a large number of patients to be screened in order to perform a clinical trial. Accruing many patients for this purpose appears to be problematic, as the cost for high-throughput genomic profiling to identify patients carrying particular mutations is still relatively high. However, with the advent of NGS technologies and prices of this innovative approach constantly decreasing, it has now become easier than ever to incorporate molecular diagnostics into clinical routine [50].

Nevertheless, the cost of molecular profiling accounts for a very small amount of the whole therapy. Molecular-guided treatment still represents the main cost driver, accounting for more than 50% of all costs [51]. Undeniably, the high costs associated with molecular profiling and targeted therapies, and limited drug access represent more barriers for successful translation of precision oncology into clinical breast cancer practice [52,53]. In our study, the cost of the recommended targeted therapy was one of the most common reason why patients did not receive the recommended treatment. Unfortunately, with the rising number of approved targeted drugs, their costs have increased during the same time [54].

As shown in this study, clinical trials unfortunately often remain unavailable for patients, mainly because of deterioration of patients’ physical condition or existing exclusion criteria for a given trial. Considering the fact that breast cancer accounts for one of the highest uses of targeted therapies, we need to find a way to ensure access to targeted therapies for patients with actionable mutations. One possible solution is to develop basket trials, testing the effectiveness of a single drug against a molecular alteration in various cancer entities. Another option is to create umbrella trials, which focus on the effect of different drugs targeting different gene alteration in a single cancer entity [55]. MTBs could serve as a platform to improve access to targeted therapies by constantly reviewing relevant clinical trial options for particular groups of patients. As other authors already suggested, the access to a MTB increases the chance for application of genetics-guided cancer care [56]. According to the recently published 5th ESO-ESMO international consensus guidelines for advanced breast cancer (ABC 5), suitable patients, ready to participate in clinical trials of novel therapies, should undergo NGS testing in centers with relevant trial options [57].

Another major benefit of implementing MTBs into clinical care is that they also improve clinicians’ knowledge about molecular oncology [58]. The complexity of the large amounts of data generated by genomic profiling requires expert review for maximum clinical benefit. MTBs provide a system to guide clinical decision-making in precision oncology, while also training physicians who are still inexperienced in this topic and improving their confidence in understanding this new field.

However, the concept of MTBs is not fully defined, as guidelines and quality criteria are still missing, which is the reason why there are great discrepancies in outcomes of clinical trials focusing on precision oncology. Different centers tend to have different patient selection criteria and also differ in selection of multigene panels used for molecular profiling. The right time of enrolling patients into trials enabling access to precision cancer care is still a matter of debate. As seen in our cohort, patients’ disease stage at time of enrollment is of great importance for evaluating the impact of personalized treatment recommendations for cancer patients. Rapid deterioration of the physical condition was one of the main reasons why patients did not receive the recommended treatment. The median turnaround times from indication for molecular profiling to MTB case discussion are still in some cases quite long for cancer patients at a late disease stage. This suggests a need to evaluate which patient groups would benefit most from implementing of precision oncology in standard oncology care. Defining actionability of genomic alterations, providing access to clinical trials and off-label drugs and quality assurance of molecular diagnostics also seem to vary from center to center. In the constantly changing world of precision oncology, there is a need for standardizing and optimizing the work of MTBs and for developing international guidelines and real-world databases to guide clinician decision-making in precision oncology.

The precision oncology field is constantly evolving. In our clinical center, we managed to evaluate this trend over the past two and half years. Comparing the results of the presented study with those of our first study where we presented results of the first 100 patients with mBC or gynecologic malignancies, we have observed an improvement in the therapy implementation rates (16% in the presented study vs. 12.5% in the previous study), in the number of recommendations given (49% vs. 42%), and in the number of mutations found (53% vs. 48%) [59]. In addition, the number of technical problems occurring in the molecular diagnostics was significantly lower in the presented trial as compared to our earlier experiences (11% vs. 17% in our last presented study). These results demonstrate the importance and potential of developing precision oncology access programs in academic centers.

In view of our results and recently published experiences, we expect molecular profiling and molecular tumor boards to become increasingly implemented in breast cancer care over the next few years. In order to maximize clinical benefit for more patients, it is essential to optimize MTB structures, reconsider selection patient criteria for tumor molecular profiling, and to determine new biomarkers and associated targeted therapies by improving access to clinical trials. In addition, it’s important to consider using liquid biopsies for molecular profiling, a revolutionary but still limited new tool for precision medicine. As an important diagnostic tool, it has advantages such as providing representative analysis in the presence of multiple tumor foci and being less invasive compared to traditional tumor biopsy analysis, but also disadvantages such as high costs and questionable sensitivity. Within the setting of our molecular tumor board, liquid biopsies were only considered in a minor part of the patients (other tumor entities) where no recent tumor biopsy was available or performable. Reasons for this were the potential false negative rates, high analyses cost due to high sensitivity systems combined with a very low chance of health insurance reimbursement.

The presented study has several limitations. First, the cohort presented, comprising 100 mBC patients, is relatively small. Moreover, our patients already had advanced-stage disease and a therefore limited number of available, previously not implemented treatment options. Thus, it is possible that our findings may not be applicable to patients exposed to comprehensive molecular profiling and MTB discussion at an earlier disease stage. Second, defining actionable mutations is challenging and also depends on approved targeted therapies at time of case presentation. As the field of molecular oncology is rapidly evolving, the importance of specific biomarkers may vary. Third, as tumors tend to evolve during the disease course, it is possible that the molecular landscape of the tumor may have changed by the time of molecular profiling. Furthermore, some studies suggest a possibility of cancers evolving under cancer therapy [60]. As some of the tissue samples were collected prior to the last systemic treatment, this may have caused inaccuracy in the matching of targeted therapies and actionable mutations. Lastly, the presented study was not designed as a randomized controlled trial, but rather as a real-world data registry.

## 5. Conclusions

Although the number of patients is still low, our experience shows that patients with mBC may benefit from implementation of MTB recommendations based on targeted panel-guided sequencing into clinical care. MTBs have proven to be a helpful tool for patient care, as they combine clinical expertise in several oncology areas in order to improve patient outcome by providing a personalized tailored-based treatment advice. They also encourage interdisciplinary knowledge transfer and are a great platform for expanding experience in precision oncology. In order to maximize the clinical utility of precision oncology, logistical support to ease access to drugs and clinical trials is needed.

## Figures and Tables

**Table 1:** Overview of studies focusing on molecular profiling in breast cancer.

Author/Study	Tumor Entity	Enrolled Patients (n =)	MP Patients	Actionable Alterations	Implemented Therapies-n (% of Enrolled)	Results
**André et al. (SAFIR01/UNICANCER)**	breast cancer	423	299 (71%)	195 (46%)	55 (13%)	ORR: 4 patients had a PR and 9 had SD > 16 weeks (3% of all patients)
**Parker et al.**	breast cancer	43	43 (100%)	40 (93%)	17 (40%)	7 patients (16% of all patients) achieved SD or PR
**Van Geelen et al.**	breast cancer	322	234 (72%)	74 (23%)	No data	No data about implementation rate and outcome

MP = molecular profiled, PFS = progression-free survival, ORR = overall response rate, SD = stable disease, PR = partial response, n.a. = not available.

**Table 2:** List of genomic alterations Level I/II/III in breast cancer as classified by the European Society for Medical Oncology (ESMO) Scale for Clinical Actionability of Molecular Targets (ESCAT) [27].

Genomic Alterations	Prevalence
**ESCAT Level I**	
*BRCA1/2* germline mutations	4%
*ERBB2* amplifications	15–20%
Microsatellite instability-high	1%
*PIK3CA* hotspot mutations	30–40%
**ESCAT Level II**	
*AKT1^E17K^* mutations	5%
*ERBB2* hotspot mutations	4%
*ESR1* mutations	10%
**ESCAT Level III**	
*BRCA1/2* somatic mutations	3%
*ERBB3* mutations	2%
*MDM2* amplifications	1%

**Table 3:** Patient characteristics (n = 100)**.**

Patient Characteristics	n =
**Median age**	52 (range 30–82)
**Number of metastatic sites at time of presentation**	
1	25
2	39
3	20
>3	16
**Metastatic sites**	
visceral	87
bone	62
brain	21
cutaneous	11
**Number of previous therapies**	
1	6
2	26
3	13
>3	55

## Supplementary Materials

The following are available online at https://www.mdpi.com/article/10.3390/diagnostics11040733/s1, Table S1: List of Gene Targets in Oncomine Comprehensive Assay v3—161 gene panel; Table S2: List of Gene Targets in Oncomine Focus Assay—52 gene panel.

## Abbreviations

MTB	Metastatic breast cancer
PD-L1	Programmed death-ligand 1
TNBC	Triple-negative breast cancer
PARP	Poly (ADP-ribose) polymerase
gBRCA	Germline BRCA
CTC	Circulating tumor cell
LoE	Levels of evidence
GR	Grade
AGO	Arbeitsgemeinschaft Gynäkologische Onkologie (German Gynecological Oncology Group)
MTB	Molecular Tumor Board
ESCAT	ESMO Scale of Clinical Actionability
PFS	Progression-free survival
PFSr	Progression-free survival ratio
HR	Hormone receptor
ER	Estrogen receptor
PR	Progesterone receptor
HER2	Human epidermal growth factor receptor 2
NGS	Next-generation sequencing
MTA	Molecular targeted agents
OR	Overall response rate
SD	Stable disease
PR	Partial remission

## Appendix A

**#**	**Alteration Found**		**Treatment Recommendation**	**Follow-Up**
1	*PIK3CA*, *ESR1* and *TP53* mutations	*PIK3CA*-Gene: chr3: 178952085, Exon 21, c.3140A > G (NM_006218.3), p.His1047Arg	alpelisib	lost to follow up
		*ESR1*-Gene: chr6: 152332832, Exon 6, c.1138G > C (NM_001122740.1), p.Glu380Gln		
		*TP53*-Gene: chr17: 7579558, Exon 4, c.128delT (NM_000546.5), p.Leu43Ter		
2	*FGFR1*, *AR* and *CCND1* amplifications	*FGFR1*-Gene: chr8: 38271444, CNV: 9.83	1. CDK4/6 inhibitor 2. Everolimus 3. AR inhibitor	not implemented
		*AR-*Gene*:* chrX: 66776185, CNV: 8		
		*CCND1*-Gene: chr11: 69456941, CNV: 10.57		
3	*PIK3CA* and *ESR1* mutations; TBL1XR1-PIK3CA gene fusion	External analysis	1. alpelisib 2. everolimus	implemented
4	*PIK3CA* mutation	*PIK3CA*-Gene: chr3: 178936091, Exon 10, c. 1633G>A (NM_006218.3), p.Glu545Lys	alpelisib	implemented
5	*ERBB2* amplification	External analysis	HER2 inhibitor	not implemented
6	*CCND1* amplification	*CCND1*-Gene: 11q13.3, chr11: 69456941, CNV: 9.3	1. CDK4/6 inhibitor 2. palbociclib + fulvestrant 3. everolimus	implemented
7	*PIK3CA*, *PTEN* mutations and *AKT3* amplification	*PIK3CA*-Gene: chr3: 178936082, Exon 10, c. 1624G > A (NM_006218.3), p.Glu542Lys	mTOR inhibitor	implemented
		*PTEN-*Gene: chr10: 89720803, Exon 8, c.955_956insA, (NM_000314.6 p.Thr319fs		
		*AKT3*-Gene: 1q43q44, chr:1: 243662992, CNV: 5.9		
8	*FGFR1* and *AKT2* amplifications; *TP53* mutation	FGFR1-Gene: 8p11.23, chr8: 38271114, CNV: 15.33	FGF1 inhibitor	not implemented
		AKT2-Gene: 19q13.2, chr19: 40739755, CNV: 11.61		
		TP53-Gene: chr17: 7579320, Exon 4, c.365_366delTG (NM_000546.5), p.Val122fs		
9	*ERBB2* mutation	*ERBB2*-Gene: Exon 19, chr17: 37880220, c.2264T > C (NM_004448.3), p.Leu755Ser	afatinib/neratinib	not implemented
10	*PTEN* deletion	*PTEN*-Gene: Exon 8, chr10: 89720798, c.955_958delACTT (NM_000314.4), p.Thr319Ter	1. IPATunity130 trial (NCT03337724) 2. exemestane + everolimus	not implemented
11	*PIK3CA* mutation	*PIK3CA-*Gene: chr3: 178936091, Exon 10, c. 1633G > A (NM_001127500.2), p.Glu545Lys	everolimus	not implemented
12	*FGFR*1, *FGF19* and *FGF3* mutations	*FGFR1*-Gene: 8p11.23, chr8: 38271114, CNV: 24.97	1. mTOR inhibitor 2. pazopanib	implemented
		*FGF19*-Gene: 11q13.3, chr11: 69513954, CNV: 19.73		
		*FGF3*-Gene: 11q13.3, chr11: 69624976, CNV: 12.97		
13	*MET* mutation	*MET*-Gene: Exon 14, chr7: 116411990, c.3029C > T (NM_001127500.1), p.Thr1010Ile	crizotinib	not implemented
14	*MYC*, *FGFR1* and *CCND1* amplifications	*MYC*-Gene: chr8: 128748884, CNV: 18.8	everolimus	implemented
		*FGFR1-*Gene: chr8: 38271444, CNV: 20.13		
		*CCND1*-Gene: chr11: 69456941, CNV: 38.33		
15	*BRCA1* mutation; *AR* amplification	*BRCA1* (external analysis)*AR*-Gene: chrX: 66776185, CNV: 7.87	1. trial (NCT01945775) 2. trial (NCT02163694) 3. bicalutamide/tamoxifen	not implemented
16	*PIK3CA* mutation	*PIK3CA*-Gene: Exon21, chr3: 178952074, c. 3129G > A (NM_006218.2), p.Met1043Ile	1. SOLAR-1 trial 2. IPATunity130 trial 3. everolimus	not implemented
17	*AKT2* amplification; *SF3B1* mutation	*AKT2*-Gene: 19q13.2, chr19: 40739755, CNV: 5.44	everolimus + hormone therapy	not implemented
		*SF3B1*-Gene: chr2: 198266834, Exon 15, c.2098A > G (NM_012433.3), p.Lys700Glu		
18	*PIK3CA* mutation; *MET* amplification	*PIK3CA*-Gene: chr3: 178952085, Exon 21, c. 3140A > G (NM_006218.3), p.His1047Arg	crizotinib	not implemented
		*MET*-Gene: 7q31.2, chr7: 116339592, CNV: 4.61		
19	*FGFR1* amplification	*FGFR1*-Gene: 8p11.23, chr8: 38271114, CNV: 9.6	1. pazopanib 2. MASTER trial	not implemented
20	*ESR1* and *PALB2* mutations; ESR1-CCDC170 fusion	*ESR1*-Gene: chr6: 152419923, Exon 9, c.1610A > C (NM_001122740.1), p.Tyr537Ser	1. platin-based chemotherapy 2. olaparib	not implemented
		*PALB2-*Gene: chr16: 23641065, Exon 5, c.2409_2410insAC (NM_024675.3), p.Ser804fs		
		*ESR1-CCDC170* fusion: chr6: 151894309, t(6;6) (q25;q25), ESR1(Ex2)-CCDC170(Ex6)		
21	*FGFR1* and *MYC* amplifications; *TP53* mutation	*FGFR1*-Gene: 8p11.23, chr8: 38271114, CNV: 19.83	1. FGFR1 inhibitor 2. mTOR inhibitor	not implemented
		*MYC*-Gene: 8q24.21, chr8: 128748724, CNV: 12.17		
		*TP53*-Gene: chr17: 7578500, Exon 5, c.423_432delinsCA (NM_00546.5), p.Pro 142fs		
22	*ERBB2* amplification	*ERBB2*-Gene: chr17: 37868125, CNV: 24.98	lapatinib, trastuzumab emtansine and pertuzumab	not implemented
23	*ARID1A* and *PIK3CA* mutations	*ARID1A*-Gene: chr1: 27023060, Exon 1, c.166C > T (NM_006015.5), p.Gln56Ter	everolimus	implemented
		*PIK3CA*-Gene: chr3: 178936091, Exon 10, c.1633G > A (NM_006218.3), p.Glu545Lys		
24	*ESR1* mutation	*ESR1*-Gene: chr6: 152419926, Exon 9, c.1613A > G (NM_001122740.1), p.Asp538Gly	fulvestrant + everolimus	not implemented
25	*FGFR1*, *CCND1*, *FGF19* and *IGF1R* amplifications; *ATM* mutation	*FGFR1*-Gene: 8p11.23, chr8: 38271114, CNV: 10.33	mTOR inhibitor	implemented
		*CCND1*-Gene: 11q13.3, chr11: 69455972, CNV: 7.54		
		*FGF19*-Gene: 11q13.3, chr11: 69513954, CNV: 7.95		
		*IGF1R*-Gene: 15q26.3, chr15: 99192814, CNV: 27.06		
		*ATM*-Gene: chr11: 108190701, Exon 44, c.6370_6371insT (NM_000051.3), p. Tyr2124fs		
26	*TP53* mutation; *FGFR1*, C*CND1*, *FGF19* und *FGF3* amplifications	*TP53*-Gene: chr17: 7577121, Exon 8, c.817C > T (NM_000546.5), p.Arg273Cys	FGFR1 inhibitor	not implemented
		*FGFR1*-Gene: 8p11.23, chr8: 38271114, CNV: 20.63		
		*CCND1*-Gene: 11q13.3, chr11: 69455972, CNV: 8.37		
		*FGF19*-Gene: 11q13.3, chr11: 69513954, 1 CNV: 0.93		
		*FGF3*-Gene: 11q13.3, chr11: 69624976, CNV: 15.77		
27	PIK3CA, ERBB2, CDKN2A mutations	*PIK3CA*-Gene: chr3:178936091, Exon 10, c.1633G > A (NM_006218.3), p.Glu545Lys	1. TDM1 + alpelisib 2. neratinib 3. CDK4/6 inhibitor 4. everolimus; in combination with trastuzumab	not implemented
		*ERBB2*-Gene: chr17: 37880219, Exon 19, c.2264T > C (NM_004448.3), p.Leu755Ser		
		*CDKN2A*-Gene: chr9:21974748, Exon 1, c.79G > T (NM_001195132.1, p.Glu27Ter		
28	TPM3(7)-NTRK1(10) fusion	*TPM3(7)-NTRK1(10)*-Gene: Exon 7 (I), chr1: 154142875-chr1: 156844362	trial (NCT02568267)	not implemented
29	*MET* mutation	*MET*-Gene: Exon 2, chr7: 116411990, c.3029C > T (NM_001127500.1), p.Thr1010Ile	cabozantinib	not implemented
30	*KRAS* and *PIK3CA* mutations	*KRAS*-Gene: chr12: 25398284, Exon 2, c.34G > C (NM_033360.3), p.Gly12Arg	peg-Doxorubicin/bevacizumab und temsirolimus/everolimus	not implemented
		*PIK3CA*-Gene: chr3: 178936082, Exon 10, c.1624G > A (NM_006218.3), p.Glu542Lys		
		*PIK3CA*-Gene: chr3: 178938934, Exon 14, c.2176G > A (NM_006218.3), p.Glu726Lys		
31	*AR* and *PIK3CA* mutations	*AR*-Gene: chrX: 66941751, Exon 6, c.2395C > G (NM_000044.3), p.Gln799Glu	everolimus	not implemented
		*PIK3CA*-Gene: chr3: 178936091, Exon 10, c.1633G > A (NM_006218.3), p.Glu545Lys		
32	*MET*, *CCND1*, *FGF19*, *FGF3* amplifications	*MET*-Gene: 7q31.2, chr7: 116339592, CNV: 4.66	*FGF1* inhibitor	implemented
		*CCND1*-Gene: 11q13.3, chr11: 69455972, CNV: 20.87		
		*FGF19*-Gene: 11q13.3, chr11: 69513954, CNV: 18.61		
		*FGF3*-Gene: 11q13.3, chr11: 69624976, CNV: 18.49		
33	FGFR1, CCND1, EGFR, PIK3CA und PDGFRA amplifications	External analysis	pazopanib	not implemented
34	*PIK3CA* mutation; *ERBB2* amplification	External analysis	1. *HER2* inhibitor 2. *HER2* inhibitor + neratinib 3. *PIK3CA* inhibitor	not implemented
35	*ERBB2* mutation; *CCNE1*, *AKT2*, *ERBB2* amplifications	*ERBB2*-Gene: chr17: 37868208, Exon 8, c.929C > A (NM_004448.3), p.Ser310Tyr	trastuzumab + lapatinib	implemented
		*CCNE1*-Gene: 19q12, chr19: 30303882, CNV: 5.51		
		*AKT2*-Gene: 19q13.2, chr19: 40739755, CNV: 6.09		
		*ERBB2*-Gene: 17q12, chr17: 37868168, CNV: 8.46		
36	*ESR1* and *PIK3CA* mutations	*ESR1*-Gene: chr6: 152419922, Exon 9, c.1609T > A (NM_001122740.1), p.Tyr537Asn	1. trial (NCT03056755) 2. everolimus	not implemented
		*PIK3CA*-Gene: chr3: 178936091, Exon 10, c.1633G > A (NM_006218.3), p.Glu545Lys		
37	*p16* high expression and *MYC* amplification	*MYC*-Gene: chr8: 128748884, CNV: 5.61	checkpoint inhibitors	implemented
38	*AKT3* amplification and *TP53* mutation	*AKT3*-Gene: 1q43q44, chr1: 243662992, CNV: 6.04	MASTER trial	not implemented
		*TP53*-Gene: chr17: 7578189, Exon 6, c.660T > A (NM_000546.5), p.Tyr220Ter		
39	*AR* amplification	*AR*-Gene: chrX: 66776185, CNV: 7.65	*AR* inhibitors	not implemented
40	*AKT1* mutation	*AKT1*-Gene: chr14: 105246551, Exon 3, c.49G > A (NM_001014431.1), p.Glu17Lys	1. *AKT* inhibitors 2. IPATunity130 trial (NCT03337724) 3. everolimus	not implemented
41	*SLX4* mutation; *FGFR1*, *CCND1*, *FGF19*, *FGFR3* amplifications	*SLX4*-Gene: chr16: 3640038, Exon 12, c.3601C > T (NM_032444.3), p.Gln1201Ter	pazopanib	implemented
		*FGFR1*-Gene: 8p11.23, chr8: 38271114, CNV: 15.4		
		*CCND1*-Gene: 11q13.3, chr11: 69455972, CNV: 5.83		
		*FGF19*-Gene: 11q13.3, chr11: 69513954, CNV: 6.01		
		*FGF3*-Gene: 11q13.3, chr11: 69624976, CNV: 6.05		
42	*ESR1* mutation	*ESR1*-Gene: Exon 9, chr6: 152419919, c.1606_1608delCTCinsAAA (NM_001122740.1), p.Leu536Lys	fulvestrant + CDK4/6 inhibitors	not implemented
43	*CCND1* and *FGF19* amplifications; *AKT1* mutation	CCND1-Gene: 11q13.3, chr11: 69455972, CNV: 9.13	1. IPATunity130 trial (NCT03337724) 2. mTOR inhibitor	not implemented
		*FGF19*-Gene: 11q13.3, chr11: 69513954, CNV: 9.99		
		*AKT1*-Gene: chr14:105246551, Exon 3, c.49G > A (NM_001014431.1), p.Glu17Lys		
44	*PIK3CA* and *TP53* mutations	*PIK3CA*-Gene: chr3: 178952085, Exon 21, c.3140A > G (NM_006218.3), p.HIS1047Arg	alpelisib	implemented
		*TP53*-Gene: chr17: 7577538, Exon 7, c.743G > A (NM_000546.5), p.Arg248Gln		
45	*CCND1* and *FGFR1* amplifications	External analysis	1. everolimus + hormone therapy 2. dovitinib	not implemented
46	*PIK3CA* and *ERBB2* mutations; *ERBB2* high expression	*PIK3CA*-Gene: chr3: 178927980, Exon 8, c.1258T > C (NM_006218.3), p.Cys420Arg	1. dual HER2 inhibitors 2. Neratinib	implemented
		*ERBB2*-Gene: chr17: 37881000, Exon 20, c.2329G > T (NM_004448.3), p.Val777Leu		
47	*FGFR1* amplification	*FGFR1*-Gene: 8p11.23p11.22, chr8: 38271444, CNV: 6.61	everolimus + hormone therapy	implemented
48	*CCND1* amplification	CCND1-Gene: 11q13.3, chr11: 69456942, CNV: 5.48	exemestane + everolimus	not implemented
49	*CCND1* and *FGFR1* amplifications	*CCND1*-Gene: 11q13.3, chr11: 69456941, CNV: 20.18	1. exemestane + everolimus 2. trial (NCT03517956)	implemented
		*FGFR1*-Gene: 8p11.23p11.22, chr8: 38271444, CNV: 28.5		
