# Supplementary material for: Implementation of Precision Oncology for Patients with Metastatic Breast Cancer in an Interdisciplinary MTB Setting
Source: Diagnostics (Basel). 2021 Apr 20;11(4):733. doi: 10.3390/diagnostics11040733 (PMC8074310; doi:10.3390/diagnostics11040733)
Supplement: Supplementary file 1 [file diagnostics-11-00733-s001.zip › diagnostics-1109530-supplementary.pdf]

diagnostics

Supplementary File

Implementation of Precision Oncology for Patients with Metastatic Breast Cancer in an Interdisciplinary MTB Setting

Elena Sultova [1], C. Benedikt Westphalen [2], Andreas Jung [3], Joerg Kumbrink [3], Thomas Kirchner [3], Doris Mayr [3], Martina Rudelius [3], Steffen Ormanns [3], Volker Heinemann [2], Klaus H. Metzeler [2], Philipp A. Greif [2], Anna Hester [1], Sven Mahner [1,4], Nadia Harbeck [1,5] and Rachel Wuerstlein [1,4,5,*]

Table S1. List of Gene Targets in Oncomine Comprehensive Assay v3—161 gene panel.

**Hotspot Genes**					
AKT1	*CTNNB1*	*GATA2*	*KNSTRN*	*NFE2L2*	*RHOA*
AKT2	*DDR2*	*GNA11*	*KRAS*	*NRAS*	*ROS1*
AKT3	*EGFR*	*GNAQ*	*MAGOH*	*NTRK1*	*SF3B1*
ALK	*ERBB2*	*GNAS*	*MAP2K1*	*NTRK2*	*SMAD4*
AR	*ERBB3*	*H3F3A*	*MAP2K2*	*NTRK3*	*SMO*
ARAF	*ERBB4*	*HIST1H3B*	*MAP2K4*	*PDGFRA*	*SPOP*
AXL	*ERCC2*	*HNF1A*	*MAPK1*	*PDGFRB*	*SRC*
BRAF	*ESR1*	*HRAS*	*MAX*	*PIK3CB*	*STAT3*
BTK	*EZH2*	*IDH1*	*MDM4*	*PIK3CA*	*TERT*
CBL	*FGFR1*	*IDH2*	*MED12*	*PPP2R1A*	*TOP1*
CCND1	*FGFR2*	*JAK1*	*MET*	*PTPN11*	*U2AF1*
CDK4	*FGFR3*	*JAK2*	*MTOR*	*RAC1*	*XPO1*
CDK6	*FGFR4*	*JAK3*	*MYC*	*RAF1*	
CHEK2	*FLT3*	*KDR*	*MYCN*	*RET*	
CSF1R	*FOXL2*	*KIT*	*MYD88*	*RHEB*	
**Full-Length Genes**					
ARID1A	*CDKN1B*	*FBXW7*	*NOTCH1*	*PTEN*	*SETD2*
ATM	*CDKN2A*	*MLH1*	*NOTCH2*	*RAD50*	*SLX4*
ATR	*CDKN2B*	*MRE11*	*NOTCH3*	*RAD51*	*SMARCA4*
ATRX	*CHEK1*	*MSH6*	*PALB2*	*RAD51B*	*SMARCB1*
BAP1	*CREBBP*	*MSH2*	*PIK3R1*	*RAD51C*	*STK11*
BRCA1	*FANCA*	*NBN*	*PMS2*	*RAD51D*	*TP53*
BRCA2	*FANCD2*	*NF1*	*POLE*	*RNF43*	*TSC1*
CDK12	*FANCI*	*NF2*	*PTCH1*	*RB1*	*TSC3*
**Copy Number Genes**					
AKT1	*CCND2*	*ESR1*	*IGF1R*	*MYCN*	*PPARG*
AKT2	*CCND3*	*FGF19*	*KIT*	*NTRKN1*	*RICTOR*
AKT3	*CCNE1*	*FGF3*	*KRAS*	*NTRK2*	*TERT*
ALK	*CDK2*	*FGFR1*	*MDM2*	*NTRK3*	
AXL	*CDK4*	*FGFR2*	*MDM4*	*PDGFRA*	
AR	*CDK6*	*FGFR3*	*MET*	*PDGFRB*	
BRAF	*EGFR*	*FGFR4*	*MYC*	*PIK3CB*	
CCND1	*ERBB2*	*FLT3*	*MYCL*	*PIK3CA*	
**Gene Fusions (Inter- and Intragenic)**					
AKT2	*ERBB2*	*FGFR3*	*NF1*	*PDGFRB*	*RELA*

ALK	*ERBB4*	*FGR*	*NOTCH1*	*PIK3CA*	*RET*
AR	*ERG*	*FLT3*	*NOTCH4*	*PRKACA*	*ROS1*
AXL	*ESR1*	*JAK2*	*NRG1*	*PRKACB*	*RSPO2*
BRCA1	*ETV1*	*KRAS*	*NTRK1*	*PTEN*	*RSPO3*
BRCA2	*ETV4*	*MDM4*	*NTRK2*	*PPARG*	*TERT*
BRAF	*ETV5*	*MET*	*NTRK3*	*RAD51B*	
CDKN2A	*FGFR1*	*MYB*	*NUTM1*	*RAF1*	
EGFR	*FGFR2*	*MYBL1*	*PDGFRA*	*RB1*	

Table S2. List of Gene Targets in Oncomine Focus Assay—52 gene panel.

**Hotspot Genes**			
ABL1	*ERBB2*	*GNAQ*	*MYC*
AKT1	*ERBB4*	*HRAS*	*MYCN*
AKT3	*ERBB3*	*IDH1*	*NRAS*
ALK	*ERG*	*IDH2*	*NTRK1*
AR	*ESR1*	*JAK1*	*NTRK2*
AXL	*ETV1*	*JAK2*	*NTRK3*
BRAF	*ETV4*	*JAK3*	*PDGFRA*
CCND1	*ETV5*	*KIT*	*PIK3CA*
CDK4	*FGFR1*	*KRAS*	*PPARG*
CDK6	*FGFR2*	*MAP2K1*	*RAF1*
CTNNB1	*FGFR3*	*MAP2K2*	*RET*
DDR2	*FGFR4*	*MET*	*ROS1*
EGFR	*GNA11*	*MTOR*	*SMO*
